# Celiac Disease Spectrum Among Patients With Iron Deficiency Anemia: A Retrospective Study From a Tertiary Care Center in Morocco

**DOI:** 10.7759/cureus.113850

**Published:** 2026-08-03

**Authors:** Yasmine El Ouafa, Monsif Fadi, Nabiha El Abdi, Houda Benrahma, Nouama Bouanani

**Affiliations:** 1 Center for Doctoral Studies (CeDoc), Interdisciplinary Laboratory of Biothechnology and Health, Mohammed VI University of Health Sciences (UM6SS), Casablanca, MAR; 2 Center for Doctoral Studies (CeDoc), Oncopathology, Cancer Biology, and Environment Laboratory, Mohammed VI Faculty of Medicine, Mohammed VI University of Health Sciences (UM6SS), Casablanca, MAR; 3 Interdisciplinary Laboratory of Biotechnology and Health, Mohammed VI Higher Institute of Biosciences and Biotechnology, Mohammed VI University of Health Sciences (UM6SS), Casablanca, MAR; 4 Hematology, Faculty of Medicine, Mohammed VI University of Health Sciences (UM6SS), Casablanca, MAR

**Keywords:** celiac disease (ced), duodenal biopsy in malabsorption syndrome, gluten-free diet, hematological manifestation, iron deficiency anemia (ida)

## Abstract

Background

Celiac disease is a chronic immune-mediated enteropathy triggered by gluten in genetically susceptible individuals. Its presentation is variable and may range from classic malabsorptive symptoms to atypical or extraintestinal manifestations. Iron deficiency anemia (IDA) is a common extraintestinal presentation and may occur without gastrointestinal symptoms, which can delay diagnosis.

Objective

To determine the hospital-based proportion of celiac disease among patients with iron deficiency anemia at Cheikh Khalifa International University Hospital, Casablanca, Morocco, and to describe their clinical, biological, serological, and histological characteristics.

Methods

We conducted a retrospective, descriptive, single-center study of patients with IDA followed in the Department of Medical Hematology between January 2020 and September 2025. Among 635 patients with IDA, 97 underwent anti-tissue transglutaminase IgA (anti-tTG IgA) testing, and 40 underwent duodenal biopsy based on clinical suspicion. Celiac disease was confirmed by positive anti-tTG IgA serology and compatible duodenal histology. One patient with strongly positive serology and normal histology was classified as having potential celiac disease according to the Oslo definitions. Data were collected retrospectively from medical records and hospital databases.

Results

Among the 635 patients with IDA, 97 underwent serological testing, of whom 18 were positive, while 40 underwent duodenal biopsy. Seventeen patients had biopsy-confirmed celiac disease, and one additional patient was classified as having potential celiac disease, resulting in an 18-patient celiac disease spectrum cohort and a crude hospital-based proportion of 2.83%. This figure reflects selective, clinically driven testing and should not be extrapolated to the general population or to unselected patients with IDA. All 18 patients were female, with a mean age of 29.9 years. Asthenia was reported in all patients, and intestinal discomfort was the most frequent digestive symptom (83.3%). Weight loss, chronic diarrhea, and steatorrhea were reported in 66.6%, 44.4%, and 27.7% of patients, respectively. Extra-intestinal manifestations included arthralgia (38.9%), bone pain (27.7%), oral aphthous ulcers (11.1%), amenorrhea (22.2%), and recurrent miscarriage (16.7%). Mean hemoglobin was 9.9 ± 1.4 g/dL, and 94.4% of patients had ferritin levels below 15 ng/mL. Among the nine patients assessed for micronutrient status, folate and vitamin D deficiencies were found in 44.4% and 66.7%, respectively. Anti-tTG IgA was positive in all 18 patients, and Marsh III lesions were found in 66.6% of cases. Gastritis and *Helicobacter pylori *infection were each identified in approximately half of the patients who underwent upper endoscopy. All patients started a gluten-free diet; 12 received oral iron supplementation, and six received intravenous iron.

Conclusion

Celiac disease may be an underrecognized cause of iron deficiency anemia in this single-center hospital cohort, particularly among young women. Celiac serology should be considered in the evaluation of unexplained or persistent IDA, including in patients without overt gastrointestinal symptoms, to facilitate earlier diagnosis and appropriate nutritional management.

## Introduction

Celiac disease is a chronic, immune-mediated enteropathy triggered by dietary gluten - a storage protein found in wheat, barley, and rye - in genetically susceptible individuals. The diagnosis rests on a combination of a specific serological profile, principally immunoglobulin A (IgA) antibodies against tissue transglutaminase and endomysium, and characteristic small-intestinal histological changes, including villous atrophy, crypt hyperplasia, and intraepithelial lymphocytosis [1]. Gluten-derived peptides, once deamidated by tissue transglutaminase, are presented to CD4+ T lymphocytes via HLA-DQ2 or HLA-DQ8 molecules, triggering the adaptive immune response that drives mucosal injury [2].

Celiac disease affects roughly 1% of the general population, although most cases remain undiagnosed because of its broad and heterogeneous clinical spectrum [3]. The classic presentation - chronic diarrhea, abdominal distension, weight loss, and growth failure in children - has become comparatively uncommon in current practice. Atypical, paucisymptomatic, or purely extra-intestinal presentations now predominate, and this shift contributes to a diagnostic delay that still frequently exceeds a decade [4,5].

Among the extra-intestinal manifestations, iron deficiency anemia (IDA) occupies a prominent place, reported in 32% to 69% of adults at diagnosis and, in roughly half of these cases, as the only detectable abnormality [6]. The underlying mechanism is principally malabsorptive: dietary iron is absorbed predominantly in the proximal duodenum and jejunum, the intestinal segment most severely damaged by villous atrophy in celiac disease [7]. Chronic low-grade mucosal inflammation, occult gastrointestinal blood loss, and coexisting folate or vitamin B12 deficiency causing a mixed or megaloblastic picture can further contribute to the anemia [8].

Iron deficiency anemia is itself among the most common hematologic conditions worldwide and a frequent reason for consultation across general medicine, pediatrics, hematology, and gastroenterology. While chronic blood loss and inadequate dietary intake account for most cases, intestinal malabsorption - with celiac disease foremost among its causes - remains an underrecognized contributor [9]. Conversely, when adults with unexplained iron deficiency anemia are systematically screened, histologically confirmed celiac disease is found in approximately 5% of cases, a figure that rises further once serology is applied routinely rather than reserved for patients with digestive symptoms [10].

Despite this well-documented association, celiac disease is seldom included in the initial workup for iron deficiency anemia; it tends to be considered only after iron supplementation fails or once gastrointestinal symptoms are already present. This delay prolongs anemia and nutritional depletion, allows mucosal damage to progress, and increases the risk of long-term complications, including osteoporosis, infertility, neurological disease, and, rarely, enteropathy-associated T-cell lymphoma [11].

The combination of highly sensitive anti-tissue transglutaminase immunoglobulin A (anti-tTG IgA) serology and targeted screening has considerably improved detection of subclinical and atypical celiac disease, particularly among first-degree relatives of affected individuals, patients with type 1 diabetes or autoimmune thyroid disease, and those with unexplained iron deficiency anemia [12]. A positive serological result is confirmed by duodenal biopsy obtained at upper endoscopy, with lesions graded according to the internationally validated modified Marsh-Oberhuber classification [13].

Moroccan and broader North African data on celiac disease presenting as iron deficiency anemia remain limited. Most regional series have focused on pediatric populations or on the classic gastrointestinal presentation, leaving a knowledge gap regarding atypical, hematology-driven presentations of the disease in adults [14].

We undertook this study to determine the hospital-based proportion of celiac disease among patients investigated for iron deficiency anemia at Cheikh Khalifa International University Hospital, Casablanca, Morocco, and to characterize the clinical, biological, serological, and histological profile of the affected patients, with the aim of identifying features that should prompt clinicians to systematically consider celiac disease in the diagnostic workup of unexplained iron deficiency anemia.

## Materials and methods

Study design, setting, and ethics

We conducted a retrospective, observational, descriptive study in the Department of Hematology at Cheikh Khalifa International University Hospital, Mohammed VI University of Health Sciences (UM6SS), Casablanca, Morocco. Medical records of consecutive patients seen for IDA between January 2020 and September 2025 were reviewed to identify those in whom celiac disease had been confirmed.

The study protocol was reviewed and approved by the Ethics Committee of Mohammed VI University of Health Sciences (UM6SS), Casablanca, Morocco (Approval No. CE/UM6SS/34/24). The study was conducted in accordance with the Declaration of Helsinki.

Patient selection

Patients were included if they met two conditions: a confirmed diagnosis of IDA - hemoglobin below the normal range for age and sex, together with low serum ferritin and a low or normal mean corpuscular volume (MCV) - and a confirmed diagnosis of celiac disease, defined as positive anti-tTG IgA serology together with compatible duodenal histopathology on biopsy. There were no restrictions on age or sex.

Patients were excluded if another clear cause of IDA was identified, such as gastrointestinal, gynecological, or urological bleeding, or a history of frequent blood donation, or if they were already diagnosed with celiac disease before their IDA was identified.

Of the 635 patients followed for IDA during the study period, 97 underwent anti-tTG IgA serological testing, of whom 18 were seropositive; all 18 subsequently underwent duodenal biopsy. Celiac disease was diagnosed based on positive anti-tTG IgA serology together with compatible duodenal histology (Marsh ≥1); 17 of the 18 seropositive patients met this combined criterion. The remaining seropositive patient had a normal duodenal biopsy (Marsh 0) and was classified as potential celiac disease according to the Oslo definitions [1], and was retained in the celiac disease spectrum cohort given strongly positive serology, a consistent clinical and hematological picture, and current guidance that such cases fall within the celiac disease spectrum and warrant follow-up rather than exclusion [11]. In addition to these 18 seropositive patients, a further 22 patients with clinical suspicion of celiac disease (persistent or unexplained IDA, digestive symptoms, or other features suggestive of malabsorption) underwent duodenal biopsy but were not retained, owing to normal or non-compatible histology and/or negative serology, bringing the total number of biopsies performed to 40. Detailed serological results were not consistently available for all 22 patients who underwent biopsy because of clinical suspicion, and these patients were therefore not included in the final celiac disease spectrum cohort. No patient was included on the basis of clinical response to a gluten-free diet alone.

Data collection

Data were gathered retrospectively with a standardized case report form, drawing on paper and electronic medical records, hospitalization and outpatient registries, the hospital information system, and the department's electronic follow-up database. We recorded demographic characteristics, medical history, clinical presentation, laboratory results, serological and histopathological findings, treatments given, and follow-up outcomes. All data were anonymized before analysis, with each patient assigned a unique study identifier; no directly identifying information was extracted or retained.

Statistical analysis

Descriptive statistics were computed using IBM SPSS Statistics version 26 (IBM Corp., Armonk, NY, USA). Continuous variables are presented as mean ± standard deviation (SD) and range, whereas categorical variables are presented as frequencies and percentages. Because this was a retrospective descriptive case series without comparison groups, no inferential statistical tests were performed, and no p-values were calculated.

## Results

Patient flow and hospital-based proportion

**Figure 1:**
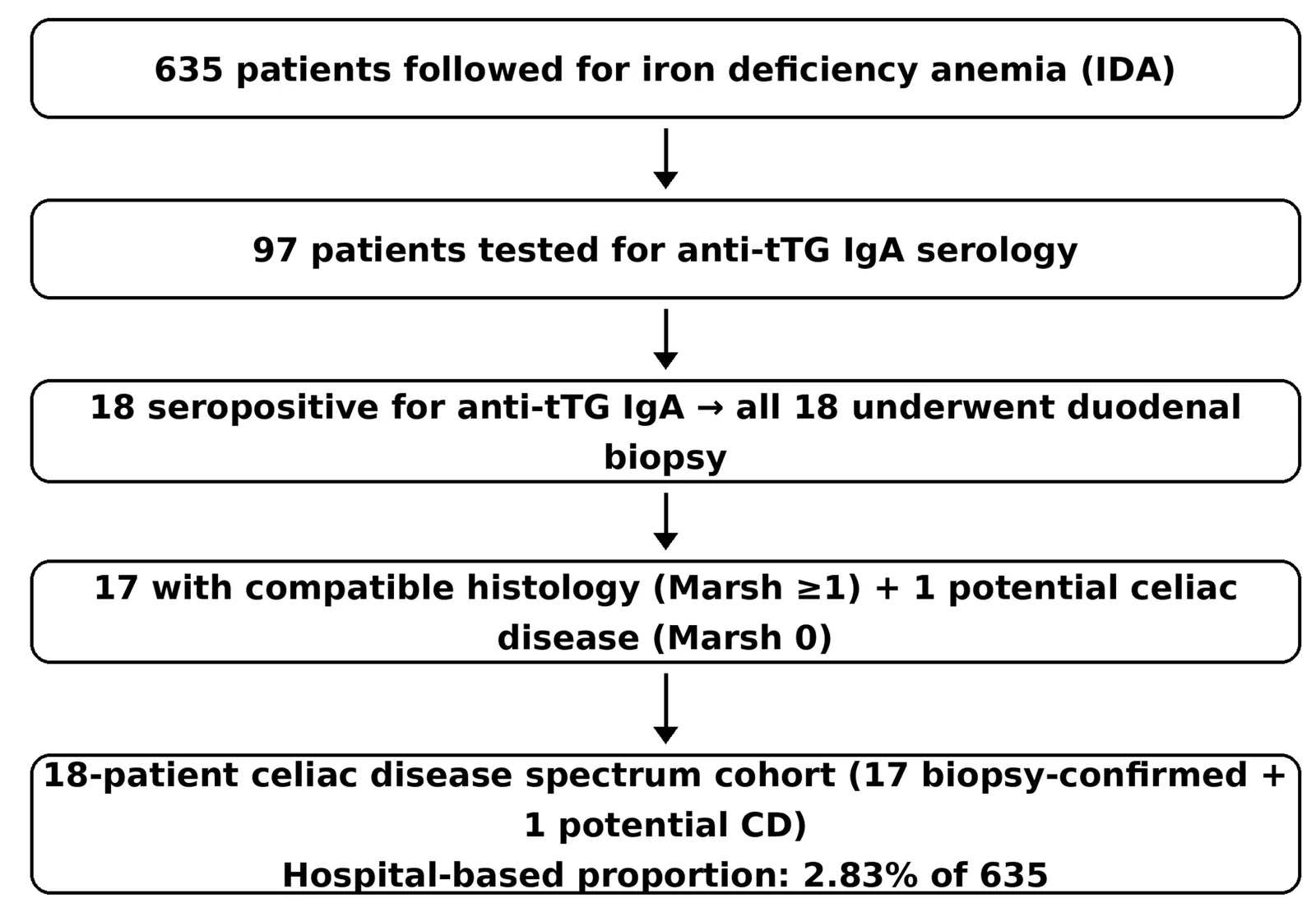
Patient flow diagram. Note: An additional 22 patients were biopsied on clinical suspicion, independently of the seropositive group above, but were not retained as celiac disease (normal or non-compatible histology and/or negative serology). Total duodenal biopsies performed: 40 (18 retained + 22 not retained). anti-tTG IgA: anti-tissue transglutaminase immunoglobulin A; CD: celiac disease

Figure 1 summarizes the patient flow from the source IDA population to the final diagnosed cohort. Of 635 patients followed for iron deficiency anemia between January 2020 and September 2025, 97 underwent anti-tTG IgA serological testing, of whom 18 tested positive; all 18 seropositive patients subsequently underwent duodenal biopsy. Of these, 17 had histology compatible with celiac disease (Marsh ≥1) and were classified as biopsy-confirmed celiac disease, while 1 had a normal biopsy (Marsh 0) but was retained as potential celiac disease per the Oslo definitions [1], given strongly positive serology and a consistent clinical picture. Together, these 17 biopsy-confirmed patients plus the 1 potential celiac disease patient constitute an 18-patient celiac disease spectrum cohort, corresponding to a crude hospital-based proportion of 2.83% among all IDA patients followed (Table 1). A further 22 patients were biopsied on clinical suspicion independently of this seropositive group but were not retained, owing to normal or non-compatible histology and/or negative serology, bringing the total number of duodenal biopsies performed to 40. Detailed serological results were not consistently available for all 22 patients who underwent biopsy because of clinical suspicion, and these patients were therefore not included in the final celiac disease spectrum cohort.

**Table 1:** Hospital-based proportion of celiac disease among patients with iron deficiency anemia. Data are presented as numbers (n) and percentages (%). No inferential statistical analyses were performed because this was a descriptive retrospective study. anti-tTG IgA: anti-tissue transglutaminase immunoglobulin A.

Category/Modality	n	%
Patients followed for iron deficiency anemia	635	-
Patients tested for anti-tTG IgA serology	97	-
Patients seropositive for anti-tTG IgA	18	-
Total patients who underwent duodenal biopsy	40	-
of whom biopsy-confirmed celiac disease	17	-
of whom potential celiac disease (Oslo criteria)	1	-
Total celiac disease spectrum cohort	18	2.83%

Demographic and medical history characteristics

The mean age of the study population was 29.9 years, ranging from 11 to 47 years. More than half of the patients (55.6%) were older than 30 years, while 27.8% were younger than 20 years and 16.7% were aged between 20 and 30 years. All 18 patients included in the study were female.

One-third of patients (33.3%) had no relevant personal history. Gastritis was the most frequent comorbidity (55.6%), followed by obesity and thyroid dysfunction (11.1% each). Type 2 diabetes and hypertension were each found in a single patient (5.6%). As several patients had more than one comorbidity, percentages do not sum to 100%.

A family history of celiac disease was reported in 22.2% of patients, while the majority (77.8%) had no relevant family history (Table 2). 

**Table 2:** Demographic and medical history characteristics.

Variable	n	%
Mean age: 29.9 years (range: 11-47)	18	—
Age < 20 years	5	27.8
Age 20-30 years	3	16.7
Age > 30 years	10	55.6
Female sex	18	100
No relevant personal history	6	33.3
Gastritis (personal history)	10	55.6
Obesity	2	11.1
Thyroid dysfunction	2	11.1
Type 2 diabetes	1	5.6
Hypertension	1	5.6
Family history of celiac disease - present	4	22.2
Family history of celiac disease - absent	14	77.8

Clinical manifestations

Asthenia was present in all patients (100%) and was the most consistent clinical sign. Pallor of the skin and mucous membranes was noted in 61.1% of cases, while muscle cramps and hair loss were reported in 33.3% and 11.1% of patients, respectively.

Intestinal discomfort was the most common digestive symptom (83.3%), followed by weight loss (66.6%), abdominal pain (55.6%), chronic diarrhea (44.4%), and steatorrhea (27.7%), reflecting an underlying malabsorption syndrome.

Arthralgia was the most frequent extra-digestive manifestation (38.9%), followed by bone pain (27.7%). Gynecological disorders, including amenorrhea (22.2%) and a history of miscarriage (16.7%), were also notable. Neurological involvement, such as ataxia, was rare (5.5%) (Table 3).

**Table 3:** Clinical manifestations of celiac disease. Patients could present with more than one clinical manifestation.

Variable	n	%
Asthenia	18	100
Pallor of skin/mucous membranes	11	61.1
Muscle cramps	6	33.3
Hair loss	2	11.1
Intestinal discomfort	15	83.3
Weight loss	12	66.6
Abdominal pain	10	55.6
Chronic diarrhea	8	44.4
Steatorrhea	5	27.7
Arthralgia	7	38.9
Bone pain	5	27.7
Amenorrhea	4	22.2
History of miscarriage	3	16.7
Oral aphthosis	2	11.1
Ataxia	1	5.5

Biological findings

Anemia was predominantly moderate, observed in 72.2% of patients, while severe and mild anemia were found in 16.7% and 11.1% of cases, respectively. The mean hemoglobin level was 9.9 ± 1.4 g/dL (range: 7.0-12.6 g/dL), according to WHO classification.

Microcytosis (MCV < 80 fL) was present in 55.6% of patients, confirming the predominantly microcytic character of the anemia, while 44.4% had a normal MCV.

Mean corpuscular hemoglobin concentration (MCHC) values were mainly distributed between 30 and 31 g/dL (38.9%), with hypochromia (MCHC < 30 g/dL) noted in a smaller subset of patients, reflecting variable degrees of iron-deficient erythropoiesis.

Ferritin levels were markedly low across the cohort. Values between 5 and 10 ng/mL were the most frequent (44.4%), while severe depletion (< 5 ng/mL) was found in 33.3% of patients, confirming profound iron deficiency.

Serum folate and vitamin D levels were not systematically assessed in all patients but were measured when clinically indicated as part of the nutritional assessment or when micronutrient deficiency or malabsorption was suspected; these data were therefore available for nine patients only. Among the nine patients tested, folate deficiency was identified in 44.4%, indicating an associated nutritional deficiency secondary to intestinal malabsorption.

Vitamin D3 deficiency was found in 66.7% of the nine patients tested, while only 22.2% had normal levels, highlighting the nutritional impact of malabsorption.

Anti-tTG IgA antibodies were positive in all patients. Half of the cohort (50.0%) showed moderate positivity (50-100 IU/mL), while strong positivity (> 100 IU/mL) was observed in 27.8% of cases, reflecting marked autoimmune activity (Table 4).

**Table 4:** Biological findings. Continuous variables are presented as mean ± standard deviation (range), and categorical variables as number (n) and percentage (%). Vitamin B9 and vitamin D were assessed in nine patients only. Hb: hemoglobin; MCV: mean corpuscular volume; MCHC: mean corpuscular hemoglobin concentration; anti-tTG IgA: anti-tissue transglutaminase immunoglobulin A.

Variable	n	%
Mean Hb: 9.9 ± 1.4 g/dL (range: 7.0-12.6)	18	—
Mild anemia (11-11.9 g/dL)	2	11.1
Moderate anemia (8-10.9 g/dL)	13	72.2
Severe anemia (< 8 g/dL)	3	16.7
Mean MCV: 78.7 ± 4.5 fL (range: 72.4-86.8)	18	—
Microcytosis (< 80 fL)	10	55.6
Normocytosis (≥ 80 fL)	8	44.4
MCHC < 28 g/dL	1	5.6
MCHC 28-30 g/dL	1	5.6
MCHC 30-31 g/dL	7	38.9
MCHC 31-32 g/dL	4	22.2
MCHC > 32 g/dL	5	27.8
Ferritin < 5 ng/mL	6	33.3
Ferritin 5-10 ng/mL	8	44.4
Ferritin 10-15 ng/mL	3	16.7
Ferritin ≥ 15 ng/mL	1	5.6
Folate deficiency (n = 9 tested)	4	44.4
Normal folate levels (n = 9 tested)	5	55.6
Vitamin D deficiency < 20 ng/mL (n = 9 tested)	6	66.7
Vitamin D insufficiency 20-30 ng/mL (n = 9 tested)	1	11.1
Normal vitamin D ≥ 30 ng/mL (n = 9 tested)	2	22.2
Anti-tTG IgA weak positivity 20-50 IU/mL (n = 18)	4	22.2
Anti-tTG IgA moderate positivity 50-100 IU/mL (n = 18)	9	50.0
Anti-tTG IgA strong positivity > 100 IU/mL (n = 18)	5	27.8

Histological and endoscopic findings

Histological examination revealed lesions compatible with celiac disease in 17 of the 18 diagnosed patients (94.4%), while a normal duodenal biopsy (Marsh 0) was observed in the remaining patient (5.6%) despite positive serology. This patient was classified as potential celiac disease according to the Oslo definitions [1] and retained in the celiac disease spectrum cohort, given strongly positive anti-tTG IgA serology, a consistent clinical and hematological picture, and current guidance recommending that such cases be managed within the celiac disease spectrum rather than excluded [11]; this scenario, while recognized, remains uncommon and is discussed further below.

Advanced lesions with villous atrophy (Marsh III) predominated, observed in 66.6% of patients, including Marsh IIIb in 33.3% of cases. Marsh I and Marsh II lesions were less frequent (16.7% and 11.1%, respectively).

Gastritis was identified in 56.3% of the 16 patients explored endoscopically (9/16), and *Helicobacter pylori *infection was found in 50.0% of cases, indicating a non-negligible frequency of associated gastric pathology (Table 5). This endoscopic figure is distinct from, though numerically close to, the 55.6% (10/18) reporting a personal history of gastritis noted earlier (see Table 2): the two percentages reflect different denominators and different sources of information (self-reported/documented history in the full cohort of 18 versus active endoscopic findings among the 16 patients who underwent upper endoscopy) and should not be read as the same measurement.

**Table 5:** Histological and endoscopic findings among the 18-patient celiac disease spectrum cohort (17 biopsy-confirmed celiac disease + 1 potential celiac disease). *H. pylori*: *Helicobacter pylori*

Variable	n	%
Lesions compatible with celiac disease	17	94.4
Normal biopsy (Marsh 0)	1	5.6
Marsh 0	1	5.6
Marsh I	3	16.7
Marsh II	2	11.1
Marsh III (overall)	12	66.6
Marsh IIIa	2	11.1
Marsh IIIb	6	33.3
Marsh IIIc	4	22.2
Gastritis present (n = 16 explored)	9	56.3
Gastritis absent (n = 16 explored)	7	43.7
*H. pylori *positive (n = 16 explored)	8	50.0
*H. pylori *negative (n = 16 explored)	8	50.0

Treatment and management

All 18 patients in the celiac disease spectrum cohort (17 biopsy-confirmed plus one potential celiac disease) started a gluten-free diet. Iron repletion was achieved orally in 12 patients (66.7%) and intravenously in six patients (33.3%); no patient was left without iron supplementation (Table 6).

**Table 6:** Distribution of patients according to treatment received. All patients received a gluten-free diet in combination with either oral or intravenous iron supplementation.

Category/Modality	n/18	%
Gluten-free diet	18	100.0
Oral iron supplementation	12	66.7
Intravenous iron supplementation	6	33.3

## Discussion

Iron deficiency anemia is a common, often nonspecific finding that frequently triggers a broader diagnostic workup, and celiac disease remains an underrecognized contributor in adult medicine. We discuss our findings from a small but well-characterized cohort of 18 women in light of the existing literature, considering in turn the demographic, clinical, biological, serological, and histological dimensions of our results.

Sex distribution

Every patient in this series was female. This extends, in an unusually pronounced way, a pattern already well established for celiac disease: in a meta-analysis of nearly 300,000 screened individuals, Jansson-Knodell et al. reported a pooled relative risk of 1.42 (95% CI, 1.27-1.57) for undiagnosed celiac disease in women compared with men, an imbalance that was even larger among children and largely disappeared in blood-donor cohorts [15]. This pattern likely reflects both biological susceptibility and differences in care-seeking or detection. An entirely female series is more extreme than population-based screening data would predict, and our small, hospital-based sample almost certainly amplifies, rather than reflects, a true sex-restricted disease process.

Personal and family history

A third of our patients had no notable personal history, while gastritis, present in just over half, was the most frequent association. This is consistent with the gastric mucosa findings reported by Gabrieli et al. in 213 newly diagnosed adult patients, in whom a normal gastric mucosa was found in only 19.7% of cases, the remainder showing lymphocytic (15.9%), chronic active (31.5%), or chronic inactive (32.9%) gastritis [16]; our figure of 55.6% falls comfortably within this range. The thyroid and metabolic comorbidities observed in a minority of patients (autoimmune thyroid dysfunction, type 2 diabetes, hypertension) mirror the broader pattern of autoimmune and endocrine associations described for celiac disease, although our numbers are too small for a quantitative comparison [17].

Family aggregation was apparent in 22.2% of patients, in keeping with current estimates of risk among first-degree relatives. A 2025 meta-analysis of 34 studies and more than 10,000 screened relatives by Karimzadhagh et al. reported a pooled seroprevalence of 11% and a biopsy-confirmed prevalence of 7%, rising to 23% among daughters and 14% among sisters [18]. Because our cohort was almost entirely female, a familial frequency in the low twenties is unsurprising and supports opportunistic screening of first-degree relatives - particularly female ones - once an index case has been identified.

Presenting complaint and clinical pattern

The anemic syndrome dominated the clinical picture in nearly every patient, with only one woman referred primarily for nutritional assessment rather than for anemia itself. This mirrors a broader shift in how adult celiac disease presents: in a primary care cross-sectional study, Sanders et al. found undiagnosed celiac disease in 4.7% of patients referred for anemia and 3.3% of those referred for fatigue alone, both considerably above background population prevalence [19]. Therrien et al., in their review of extraintestinal manifestations, similarly describe anemia and fatigue among the most consistently reported non-digestive presentations of adult celiac disease, alongside dermatologic, neurologic, and reproductive features [17]. Together, these observations support an anemic or paucisymptomatic presentation as at least as relevant a trigger for celiac serology as the classical malabsorptive syndrome.

Gastrointestinal and extra-digestive manifestations

Digestive complaints remained common in our series: intestinal discomfort, weight loss, and abdominal pain were each reported by more than half of patients, with chronic diarrhea or steatorrhea in a smaller subset, indicating a tangible malabsorptive component even though the entry point into care was hematologic rather than gastroenterologic. Therrien et al. and Montoro-Huguet et al. both note that classic and non-classic presentations frequently coexist in adults, particularly once anemia has already drawn attention to an underlying intestinal cause [17,20].

The extra-digestive manifestations we observed - arthralgia, bone pain, oral aphthous ulcers, and gynecological symptoms - illustrate how far celiac disease can extend beyond the gut. Joint involvement was the most frequent, reported in close to four in ten patients. A 2025 meta-analysis by Poddighe et al., covering nearly 7,000 patients with biopsy-confirmed celiac disease, found a pooled prevalence of joint complaints of 10.7% (95% CI, 6.9-15.1), rising to 16.8% in adult cohorts alone [21]; our higher figure likely reflects that patients were identified through an anemic presentation rather than unselected screening, but it confirms that joint pain belongs to the recognized clinical spectrum rather than representing an incidental finding. Oral aphthous stomatitis, present in two patients, has similarly been linked to celiac disease, with Nieri et al. reporting an odds ratio of 3.79 (95% CI, 2.67-5.39) for recurrent aphthous ulcers in celiac patients compared with controls [22]. The gynecological history in our cohort - amenorrhea in four patients and prior miscarriage in three - echoes the association between celiac disease and reproductive disorders described by Tersigni et al., whose meta-analysis found an odds ratio of 5.82 (95% CI, 2.30-14.74) for celiac disease among women with recurrent miscarriage and a relative risk of 1.39 (95% CI, 1.15-1.67) for miscarriage among women already diagnosed with the disease [23].

Hematologic and biochemical profile

Anemia in our series was predominantly moderate (mean hemoglobin, 9.9 ± 1.4 g/dL), figures that align closely with published cohorts identified through an iron deficiency anemia presentation. Among 402 Iranian patients with iron deficiency anemia, Baghbanian et al. identified celiac disease serologically in 10.4%, all with compatible duodenal histology, and found no significant difference in mean hemoglobin between celiac and non-celiac patients [24]. A Turkish prospective study by Çekin et al. reported a mean pretreatment hemoglobin of 10.3 ± 0.64 g/dL in celiac patients [25], while an Omani case-finding study using a hemoglobin threshold below 11.0 g/dL in women identified celiac disease in 7.7% of 104 adults screened for unexplained iron deficiency anemia [26]. The convergence of these figures across different populations suggests that, once celiac disease is uncovered through anemia, hematologic impairment tends to be moderate to severe rather than borderline, regardless of geography.

Microcytosis was present in just over half of our patients, with the remainder normocytic, a split broadly consistent with Montoro-Huguet et al.'s description of iron deficiency anemia in celiac disease as typically microcytic and hypochromic, while noting that red cell indices alone lack the specificity to discriminate celiac from non-celiac iron deficiency [20]. The same caveat applies to the low or borderline mean corpuscular hemoglobin concentration values observed here: Baghbanian et al. likewise found no significant difference in erythrocyte indices between celiac and non-celiac patients with iron deficiency anemia, reinforcing that hematologic parameters should raise suspicion rather than confirm the diagnosis [24]. White cell counts showed no consistent pattern in our cohort, in line with the broader literature describing celiac disease as overwhelmingly a disorder of the red cell line, with leukopenia an uncommon and usually secondary finding [6].

Ferritin was uniformly and often severely depleted, with most patients below 15 ng/mL, consistent with both the Omani screening threshold of 13 ng/mL used by Ambusaidi et al. [26] and the broader characterization by Montoro-Huguet et al. of ferritin below 30 ng/mL as a sensitive marker of iron deficiency and below 15 ng/mL as strongly suggestive of depleted iron stores in the absence of inflammation [20].

Vitamin status

Folate and vitamin D were tested in only half of our patients, yet deficiencies were frequent where testing was performed: 44.4% for folate and nearly two-thirds for vitamin D. Both findings fit a well-described pattern of micronutrient depletion at diagnosis. In a Dutch cohort of 80 newly diagnosed, untreated adult celiac patients, Wierdsma et al. found that 87% had at least one nutrient value below the lower limit of normal, including folate deficiency in 20% [27]. A more recent meta-analysis by Lamjadli et al., covering 45 studies, confirmed that both folate and vitamin D levels are significantly lower in celiac patients than in controls [28]. For vitamin D specifically, our findings also align with the Italian cohort studied by Ciacci et al., who reported a mean 25-hydroxyvitamin D level of 22.3 ng/mL in untreated celiac patients versus 28.7 ng/mL in those already on a gluten-free diet, a gap of similar magnitude to what we observed between deficient and sufficient patients [29]. These findings support systematic screening for folate and vitamin D deficiency once celiac disease is suspected, rather than reserving testing for patients with overt malabsorptive symptoms.

Serology and histology

Anti-tissue transglutaminase IgA was positive in every patient, with titers spanning weak, moderate, and strong positivity. This mirrors other cohorts identified through iron deficiency anemia: all 42 seropositive patients identified by Baghbanian et al. had compatible duodenal histology [24], and in the Omani case-finding study, two of three anti-tTG IgA-positive patients also tested positive for endomysial antibodies [26]. Histology in our series was dominated by advanced Marsh III lesions in two-thirds of patients. One patient had a normal biopsy (Marsh 0) despite strongly positive serology and was classified as potential celiac disease per the Oslo definitions [1] rather than excluded from the cohort; this recognized, if uncommon, scenario is generally attributed to early or patchy mucosal involvement and underscores why serology and histology are best interpreted jointly, alongside clinical and hematological data, rather than as interchangeable tests [20].

A complementary, evolving line of evidence suggests that in adults with markedly elevated tTG-IgA titers - at least ten times the upper limit of normal - biopsy confirmation may not always be required: a 2024 meta-analysis of 18 studies and more than 12,000 participants found this threshold to have a specificity of 100% (95% CI, 98-100%) for celiac disease, although sensitivity remained moderate at 51% [30]. All patients in our series nonetheless underwent biopsy, consistent with standard practice for adults presenting through a hematologic rather than a screening pathway. The predominance of advanced atrophic lesions in our cohort fits the broader observation that, when celiac disease is uncovered through an already-established anemia rather than through routine screening, mucosal damage has often had time to progress.

Endoscopic examination of the stomach, performed in 16 of our 18 diagnosed patients, revealed gastritis in 56.3% of cases and *Helicobacter pylori *infection in exactly half. The frequency of gastritis falls within the wide range reported in dedicated histological studies of the celiac stomach, including the Italian cohort of Gabrieli et al., in which over 80% of newly diagnosed celiac patients had some form of gastric inflammation on biopsy [16]. The relationship with *H. pylori *is less straightforward: a meta-analysis of 26 studies and more than 6,000 celiac cases by Amlashi et al. found a significant inverse association between *H. pylori *colonization and celiac disease, with a pooled odds ratio of 0.56 (95% CI, 0.45-0.70) [31]. Our finding of *H. pylori *positivity in half of the patients tested exceeds what this inverse association would predict, although the comparison is limited by our small sample size, the absence of a control group, and possible differences in local *H. pylori *prevalence.

Study limitations

This study has several limitations. Its retrospective, descriptive, single-center design relied on medical records, hospitalization registers, the hospital information system, and a locally maintained follow-up file, which carries an inherent risk of missing or incomplete data; most patients were seen only intermittently in outpatient consultation rather than through a structured follow-up program, so not every file contained a complete set of investigations.

Serological testing (97/635) and duodenal biopsy (40/635) were performed selectively, based on clinical judgment rather than systematic screening of all IDA patients; consequently, the reported crude hospital-based proportion of 2.83% should not be interpreted as the true prevalence of celiac disease among all IDA patients, nor extrapolated to the general Moroccan population. This selective testing strategy also introduces a form of selection (verification) bias: patients were referred for serology and biopsy largely because they already displayed clinical features suggestive of celiac disease, so the clinical, biological, and histological profile described here is inherently shaped by the same suspicion that triggered testing, rather than reflecting an unselected population of IDA patients; this circularity should be borne in mind when interpreting the frequency of individual clinical or biological findings within the cohort.

The sample size of confirmed cases was small (n = 18), which limits statistical power, widens the uncertainty around all reported proportions, and constrains how far these findings can be generalized. The single-center setting, at Cheikh Khalifa International University Hospital, Casablanca, is a further limitation, since diagnostic practices and management pathways can vary across institutions.

Sub-sample denominators varied across investigations depending on data availability: folate and vitamin D assays were not performed in every patient (9/18), and upper gastrointestinal endoscopy with gastric biopsy was performed in 16 of the 18 patients, reflecting the retrospective, non-protocolized nature of data collection rather than a predefined sampling strategy; these varying denominators are reported explicitly alongside each finding and should be kept in mind when comparing proportions across tables.

The specific anti-tTG IgA assay (manufacturer and reagent kit) and the laboratory reference ranges underlying the weak/moderate/strong positivity categories in Table 4 were not consistently documented in the retrospective records and could not be reported; this limits comparability with cohorts using different assay platforms and cutoffs, and future prospective studies should record this information systematically.

Structured follow-up data - gluten-free diet adherence, serological evolution, and long-term hematological recovery - were not consistently available and could not be systematically reported. Similarly, serum iron and transferrin saturation were not consistently available in the retrospective records and therefore could not be systematically reported alongside the other biological parameters in Table 4.

Despite these constraints, the study offers a coherent clinical and paraclinical picture of iron deficiency anemia revealing celiac disease in our hospital setting.

Larger, multicenter, prospective studies will be needed to enlarge sample sizes, improve representativeness, and better define where celiac disease fits in the etiological workup of iron deficiency anemia. Such studies should systematically assess clinical, biological, vitamin, serological, and histological parameters, along with structured follow-up once a gluten-free diet and iron supplementation have begun. Greater awareness among clinicians, combined with more standardized diagnostic protocols and data collection, could go a long way toward earlier detection and better care for patients whose celiac disease first comes to light through an iron deficiency.

## Conclusions

Celiac disease remains an underrecognized cause of iron deficiency anemia in this single-center hospital cohort. Because it can present in so many different ways - sometimes with anemia as the only clue - diagnosis is often delayed. In our series, an 18-patient celiac disease spectrum cohort was identified among patients investigated for iron deficiency anemia, comprising 17 patients with biopsy-confirmed celiac disease (positive serology together with compatible duodenal histology) and one patient with potential celiac disease (positive serology with a normal biopsy, per the Oslo definitions); together, this cohort corresponds to a crude hospital-based proportion of 2.83%. Patients were mostly young women presenting with anemia-related symptoms and, in many cases, digestive or extra-digestive manifestations along with additional nutritional deficiencies. These findings support routinely considering celiac disease when working up unexplained, persistent, or severe iron deficiency anemia, even without typical gastrointestinal symptoms; early recognition may facilitate timely initiation of a gluten-free diet and appropriate correction of associated nutritional deficiencies.
